# Critical care management of acute intoxications, dynamics and changes over time: a cohort study

**DOI:** 10.1007/s11739-024-03570-2

**Published:** 2024-03-19

**Authors:** Alexander Christian Reisinger, Nikolaus Schneider, Nikolaus Schreiber, Martina Janisch, Ines Rauch, Peter Kaufmann, Gerrit Wünsch, Philipp Eller, Gerald Hackl

**Affiliations:** 1 Department of Internal Medicine, Intensive Care Unit, Medical University of Graz, Auenbruggerplatz 15, 8036 Graz, Austria; 2 Department of Anesthesiology, LKH Hochsteiermark, Bruck an der Mur, Austria; 3 Institute for Medical Informatics, Statistics and Documentation, Medical University of Graz, Graz, Austria

**Keywords:** Poisoning, Intoxication, Drugs, Coma, Toxin, Extracorporeal

## Abstract

Acute intoxications and poisonings are a relevant cause for ICU admission of critically ill patients. This study aimed to determine the characteristics of intoxicated patients in a tertiary center medical ICU in Austria over time and to investigate parameters associated with ICU mortality. This study was a retrospective data analysis including adult ICU patients from the years 2007 to 2021. In addition to ICU documentation, pre-hospital, and emergency department documents as well as autopsy reports were utilized. In an exploratory subanalysis, we compared these findings to a historical dataset from our facility from 1992 to 1996. We identified 581 cases admitted to the medical ICU because of acute poisoning (2007–2021), of which 45% were female and 46.6% were mixed intoxications. Suicidal intent was the primary cause of intoxication (48.2%) and ICU length of stay was median 1.2 days. The majority of deceased patients received pre-hospital mechanical CPR. Primary and secondary poison/toxin removal modalities were used in 29.9% and 11.7% of cases, whereas antidotes were administered in 54.4%. Comparing the data with a historical cohort (*n* = 168), we found a shift in primary detoxification away from gastric lavage and an increase in alternative secondary poison/toxin removal techniques. The ICU mortality was 4.1% and 4.2% in the present and historic cohort, respectively. Pre-existing psychiatric illnesses increased from 49% in the historic to 69% in the present cohort. Psychiatric illness predisposes patients to severe intoxications necessitating ICU care, thus increasing prevention measures seems warranted. Females did present with a different spectrum of intoxications compared to males. ICU mortality remained low over time and most deceased patients had a grim prognosis already on ICU arrival.

**Supplementary Information:**

The online version contains supplementary material available at 10.1007/s11739-024-03570-2.

## Introduction

Acute intoxications following the inhalation, ingestion or intravenous administration of substances are a frequent cause for admission to medical intensive care units (ICU). Patients with acute intoxications can have mild symptoms, but may also present with life-threatening organ dysfunctions that require intensive care treatment [1]. Most patients presenting to the emergency department with intoxications can be discharged home, but around 4–15% of all patients with acute poisoning need critical care [2–4]. However, the rate of ICU admission varies vastly among countries and regions worldwide with reported ICU admission rates of up to 40% [4]. Reasons include different distributions and availability of specific substances such as organophosphates, but also the availability of infrastructure for comprehensive patient monitoring and ICU resources. Furthermore, patients may be admitted to an ICU for observation, but also for full code ICU therapies including invasive therapeutic measures such as intubation, mechanical ventilation, and different modalities of poison elimination. The reasons for intoxications vary from suicidal and self-harm intent to recreational and accidental intoxications. Information obtained from patient, family or bystanders is often unreliable. Therefore, objective clinical and laboratory parameters are necessary to evaluate the correct prognosis of patients [5–7].

The aim of the study was to describe the characteristics of intoxications in a medical ICU of an Austrian tertiary hospital over time and to investigate the parameters associated with ICU mortality in intoxicated patients admitted to ICU. A better understanding of this particular population may allow for efficient allocation of resources and careful management in times of limited ICU capacities.

## Methods

We retrospectively investigated adult (≥ 18 years of age) acute intoxicated patients admitted to the ICU of the Department of Internal Medicine at the Medical University of Graz, Austria, between 1st January 2007 and 31st December 2021. Patients admitted to ICU for other reasons were excluded from analyses. In addition, a previously unpublished historic 5-year cohort of all intoxicated patients (including intoxication type, comorbidities, primary and secondary poison/toxin removal techniques and other therapies) recruited on our medical ICU from 1992 to 1996 served as an exploratory internal comparison group. The study protocol was approved by the Institutional Review Board (IRB) of the Medical University of Graz, Austria (31–205 ex 18/19, 31–173 ex 18/19 and 34–342 ex 21/22) and complied with the Declaration of Helsinki.

As a search strategy to identify patients admitted to ICU for acute intoxication from electronic medical records and charts of all patients, we used two steps. We performed a keyword-based search strategy using German search terms (supplementary Table 1) in ICU medical documentation (“decursus morbi”). In addition, we did a code-based search of International Classification of Diseases (ICD) diagnosis codes (supplementary Table 2). Duplicates were removed from the detected results and all remaining patients manually screened to identify those admitted to ICU with intoxications. All available documents including discharge letters, decursus morbi, charts, nurse documentation, documents from ambulance services and emergency physicians, laboratory data, autopsy data, and others were included. In addition, the collection of the data was done on a case-by-case basis, meaning that patients with multiple ICU stays, due to multiple independent intoxication events, were investigated separately. On the one hand, intoxicated patients were categorized according to the underlying pharmacological group based on Brandenburg et al. [8] and on the other hand according to the circumstances leading to the intoxication (Supplementary Table 3). A mixed intoxication was defined as the intake or exposure of at least two different substances out of two different intoxication categories. An intoxication with multiple substances was specified as an intoxication with at least two substances out of the same and/or different intoxication categories. Demographic data including age, sex, date and time of admission, length of ICU stay, rate of intubation, length of mechanical ventilation, ICU mortality, and laboratory values were investigated. Primary poison/toxin removal was defined as either activated charcoal, gastric lavage, ipecac syrup-induced emesis, whole bowel irrigation, or endoscopic medication retrieval. Secondary poison/toxin removal was defined as multi-dose activated charcoal, forced diuresis defined in our study with at least 5 l of intravenous fluids and repetitive doses of diuretics, urine alkalization, lipid rescue therapy, hyperbaric oxygenation (HBO), or extracorporeal poison/toxin removal. The latter included hemodialysis, hemodiafiltration, hemoperfusion, hemofiltration, molecular adsorbent recirculation system (MARS), and plasma exchange. The actual use of antidotes was counted without critical retrospective reevaluation of their actual indication. According to Larsen et al. [9], we selected patients admitted to ICU for intoxication or poisoning, used all available medical documentation at our institution to obtain all potential data regarding the type and reason of the intoxication, demographic and laboratory variables were extracted from the electronic medical documentation, and all patients were independently checked for correct attribution after data collection and consensus reached if there may have been any ambiguity. The primary objective was to give a detailed overview of all intoxications and the associated intoxication categories and to investigate parameters associated with ICU mortality. Secondary objectives included investigation of demographic parameters and changes over time.

All statistical analyses were performed with SPSS 27 (SPS, IBM, Armonk, NY) and Stata 15.0 (Stata Corp., Houston, TX, USA). Continuous variables were summarized as medians [25th—75th percentile], and categorical variables as absolute frequencies (%). Associations between variables were computed with cross-tabulations, Mann–Whitney *U* tests, χ2 tests, Kruskal–Wallis tests, and Fisher’s exact tests, as appropriate. The prognostic associations between ICU mortality and potential baseline predictors were computed with univariable and multivariable logistic regression. Significance level was determined at 0.05.

## Results

In the investigated 15 years, we identified 581 cases of acute poisoning admitted to our medical ICU. The median age of all patients was 42 years [25th—75th percentile: 28–55] and 45% were female. In general, female intoxicated patients were older than male ones with medians of 47 and 37 years, respectively (*p* < 0.0001). The frequency of intoxication declined with increasing age. Thus, 75% of intoxicated patients were younger than 55 years. Most patients were admitted to the ICU once, but 21 patients (3.6%) were admitted twice, six patients (1%) three times, and two patients more often than that. The majority of patients (69%) had pre-existing psychiatric illnesses (Supplementary Table 4). The laboratory parameters of this cohort are shown in Table 1. While all-cause admissions to the ICU increased over time, the relative frequency of ICU admissions for toxicological reasons remained stable: 3.6% of admissions were intoxications in 2007, 1.9% in 2012 and 3.4% in 2021 (Kruskal–Wallis *p* over all years = 0.450). Admissions were independent from daytime with 57% of cases admitted from 06:00 a.m. to 06:00 p.m. and 43% admitted during nighttime. Most patients were admitted from the emergency department (64%), directly admitted to ICU from emergency medical services (24%), and rural hospitals (5%).

**Table 1:** Laboratory parameters in the present cohort

Variable	Cohort (*n* = 581)	Normal range	Missing values
Demographics			
Age (years)	42 [28–55]	N/A	0
Female sex	262 (45%)	N/A	0
Pre-existing psychiatric illness	398 (69%)	N/A	0
Intubation	231 (40%)	N/A	0
Laboratory parameters			
White blood count (G/L)	9.5 [7.2–13.0]	4.4–11.3	2
Hemoglobin (g/dL)	13.4 [12.1–14.7]	13–17.5	1
Platelets (G/L)	221 [180–269]	140–440	2
C-reactive protein (mg/L)	2.9 [1.0–11.0]	< 5.0	2
Creatinine (mg/dL)	0.9 [0.7–1.1]	< 1.2	2
Alanine transaminase (U/L)	25 [17–45]	< 35	8
Aspartate transaminase (U/L)	32 [22–57]	< 35	3
Prothrombin time INR	1.06 [0.98–1.14]	1.0	30
pH	7.39 [7.33–7.44]	7.35–7.45	122
Severity and outcomes			
Number of substances	2 [1–3]	N/A	24
ICU length of stay (days)	1.2 [0.7–2.3]	N/A	0
ICU mortality	24 (4%)	N/A	0

*ICU* intensive care unit, *INR* international normalized ratio

The most common intoxication category was the group of mixed intoxications accounting for 271 cases (46.6%) in all patients. Suicidal intent was the reason for the acute intoxication in 48.2% of all cases, 34.8% of male patients and 64.5% of female intoxicated patients (*p* < 0.0001). Recreational poisoning occurred in 26.9% of all patients; 40.8% of males and 9.9% of females. Other reasons were less common with accidental and iatrogenic poisoning accounting for 15.7% and 2.6% of all cases, respectively. Distribution of intent varied between age groups: suicidal intent was more common in patients with mid-range age (median 45 [30–55] years) and recreational intoxications occurred in younger patients (median 30 [24–39]), while in older patients (median 57 [42–72]) accidental intoxications were the most frequent cause (Kruskal–Wallis p between groups < 0.0001). Suicidal intent was also more common in patients with pre-existing psychiatric conditions compared to those without (54.0% vs 35.5%, *p* < 0.0001).

In total, around half of the combined drug intoxications included sedatives (*N* = 151/271, 55.7%) or antidepressants, antipsychotics, or anticonvulsants (*N* = 137/271, 50.6%) as an intoxicating substance, while ethanol was present in 43.9% of mixed intoxications. When considering both isolated and mixed intoxications, drugs out of the group of antidepressants, antipsychotics, or anticonvulsants were the most frequently used substances in the population presented. Multiple substances, defined as ≥ 2 substances from the same and/or different categories, were used in 330 cases (56.8%). Most patients took one substance (251 cases, 43.2%), two substances were used in 138 cases (23.8%), three in 15.3%, and more than three in 13.6%. Out of all cases, the number of substances remained unclear in 4.1%. When comparing sexes, female patients took more substances than men (*p* = 0.011). Substance category-specific urine drug tests (enzyme immunoassays) were performed in 48.2% of intoxicated patients and thereof 76.7% had at least one positive result.

### Primary and secondary poison/toxin removal

**Fig. 1:**
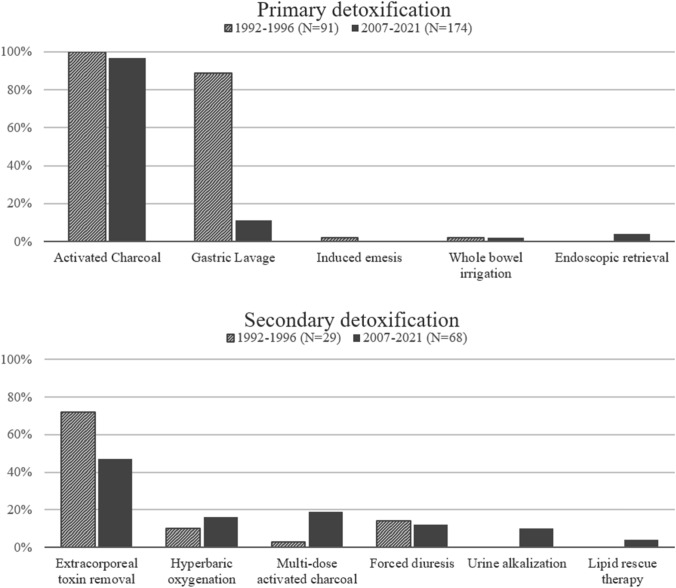
Comparison of the frequency of poison or toxin removal techniques in the historic and recent cohorts. Note that patients may have received more than one method

Primary and secondary removal techniques were used in 29.9% and 11.7% of cases, respectively. Activated charcoal was the most common method of primary detoxification and was used in 97% of patients receiving primary removal techniques. Other primary detoxification techniques were less frequently applied (Table 2, Fig. 1). Females received primary detoxification methods significantly more often than males (40.5 vs 21.3%; p < 0.0001). Dialysis, multi-dose activated charcoal, and HBO were the predominantly used secondary detoxification techniques. There were no differences in usage of secondary detoxification between females and males (11.1% vs 12.2%; *p* = 0.666). As part of secondary detoxification, extracorporeal poison or toxin removal is available in different modalities. In 18 males (5.8% of males) and 11 females (4.2% of females), dialysis was used for removal. In addition, two females received plasma exchange and one MARS was performed. Some additional patients received hemodialysis to treat complications such as acute kidney injury following rhabdomyolysis, but not for the purpose of poison or toxin removal.

**Table 2:** Primary and secondary poison or toxin removal

Subgroup: primary poison or toxin removal (*N* = 265)			
Method	Historic cohort *N* = 91 (%)	Present cohort *N* = 174 (%)	
Activated charcoal	91 (100%)	169 (97%)	0.168
Gastric lavage	89 (98%)	11 (6%)	< 0.0001
Induced emesis	2 (2%)	0 (0%)	0.117
Whole bowel irrigation	2 (2%)	2 (1%)	0.609
Endoscopic medication retrieval	0 (0%)	4 (2%)	0.302

Subgroup: secondary poison or toxin removal (*N* = 97)			
Method	Historic cohort *N* = 29 (%)	Present cohort *N* = 68 (%)	
Multi-dose activated charcoal	1 (3%)	13 (19%)	0.058
Forced diuresis	4 (14%)	8 (12%)	0.747
Urine alkalization	0 (0%)	7 (10%)	0.099
Lipid rescue therapy	0 (0%)	3 (4%)	0.552
Hyperbaric oxygenation	3 (10%)	11 (16%)	0.544
Extracorporeal removal	21 (72%)	32 (47%)	0.022

Historic cohort included patients during the years 1992–1996. New cohort included the investigated years 2007–2021. Note that patients may have received more than one method. *P* values have not been adjusted for multiple testing

### Antidotes

Antidotes were used in 316 cases (54.4%), of which flumazenil was administered most commonly, followed by naloxone. Specific and supportive antidotes were given in 57.3% of females and 52.0% of males. There was no difference in antidote use between males and females (*p* = 0.209). Examples of other antidotes given were acetylcysteine, physostigmine, digitalis antibodies, fomepizole, toluidine blue, methylene blue, silymarin, and hydroxocobalamin (Supplementary Table 5).

### Mechanical ventilation

231 (39.8%) patients needed invasive mechanical ventilation. There were no differences between female and male patients (37.8% of women and 41.4% of men; *p* = 0.379). The median duration of invasive mechanical ventilation was 18.0 [8.2–45.9] h in the whole cohort with 19.0 [7.5–47.8] and 18.0 [8.3–44.9] h in females and males (*p* = 0.857), respectively. Most patients (60.9%) were liberated from mechanical ventilation within 24 h and 77.0% were weaned within 48 h of initiation. Only 5.7% of patients received mechanical ventilation for more than 7 days.

### Length of stay, and discharge and mortality

The median length of stay in the ICU was 1.2 [0.7–2.3] days. Men stayed in the ICU for 1.2 [0.7–2.7] days, while women were treated for 1.3 [0.8–2.1] days (*p* = 0.834). 232 patients (40%) were discharged from ICU within 24 h, 71% within 48 h. Thus, treatment for more than 7 days was uncommon (6.2%). Of the 581 admissions to ICU, 74 patients (13%) were discharged directly home. Most patients (*n* = 298/581; 51%) were transferred to a psychiatric ward, whereas 155 patients (28%) were transferred to a general ward for further therapy. 18 patients were transferred to another ICU and 7 patients were transferred to a correction facility. The ICU mortality in the study population was very low at 4.1% (24 cases). Most patients who died within the ICU had a poor prognosis already on arrival. 15 of these 24 deceased patients (63%) underwent cardiopulmonary resuscitation before admission to hospital. There was no difference in the rate of mortality between male and female patients (*p* = 0.941). Parameters that were associated with mortality in univariable logistic regression analysis included intubation, but neither age, sex, LOS-ICU, pre-existing psychiatric illness, nor number of used substances (Table 3). Out of the laboratory parameters, aspartate transaminase, alanine transaminase, serum creatinine, white blood cell count, C-reactive protein, and pH were associated with ICU mortality. In multivariable logistic regression, including the variables that were significantly associated in univariable regression analyses, intubation, elevated alanine transaminases, creatinine, and low pH remained significant predictors of ICU mortality (Table 3).

**Table 3:** Association with ICU mortality in the present cohort

Variables	ICU mortality		
	Odds ratio (OR)	95%CI	*p*
Age (per 1 year increase)	1.00	0.98–1.02	0.947
Female sex	1.03	0.45–2.34	0.941
ICU LOS (per 1 day increase)	1.01	0.94–1.11	0.745
Intubation	8.20	2.77–24.32	** < 0.0001**
Pre-existing psychiatric illness	0.53	0.23–1.20	0.128
Number of substances	0.88	0.62–1.24	0.463
WBC (per 1 G/L increase)	1.11	1.04–1.19	**0.003**
Hemoglobin (per 1 g/dL increase)	1.02	0.82–1.26	0.884
Platelets (per 50 increase)	0.76	0.56–1.04	0.082
CRP (per 100 mg/L increase)	1.89	1.09–3.28	**0.024**
Creatinine (per 1 mg/dL increase)	1.56	1.22–1.99	** < 0.0001**
ALT (per 100 U/L increase)	1.05	1.02–1.08	** < 0.0001**
AST (per 100 U/L increase)	1.02	1.01–1.04	**0.005**
PT INR (per 1 unit increase)	1.39	0.94–2.07	0.101
pH (per 0.1 increase)	0.40	0.29–0.55	** < 0.0001**
Multivariable model*			
Intubation	8.60	1.85–39.78	**0.006**
WBC (per 1 G/L increase)	0.99	0.89–1.08	0.745
CRP (per 100 mg/L increase)	1.86	0.89–3.91	0.100
Creatinine (per 1 mg/dL increase)	1.42	1.01–2.00	**0.046**
ALT (per 100 U/L increase)	1.05	1.02–1.09	**0.005**
pH (per 0.1 increase)	0.42	0.29–0.62	** < 0.0001**

Statistically significant *p*-values are given in bold

AST was omitted from the model due to strong collinearity with ALT. *ICU* intensive care unit, *LOS* length of stay, *CI* confidence interval, *WBC* white blood count, *CRP C*-reactive protein, *ALT* alanine aminotransferase, *AST* aspartate aminotransferase, *PT INR* prothrombin time international normalized ratio

*452 patients (129 excluded due to missing values). We therefore performed a second multivariable model including the same variables except the pH due to the high number of missing values for this parameter. The second multivariable model included 572 patients (9 excluded for missing values) and results remained similar with significant results for intubation (*p* < 0.001), creatinine (*p* = 0.040), and ALT (*p* = 0.002), but not WBC (*p* = 0.291) or CRP (*p* = 0.214)

### Comparison with historic cohort

As an exploration of changes over time, we also compared this study population to a historic cohort that was recruited from 1992 to 1996 at our institution. Thereby, we identified 168 patients who were admitted to ICU because of an acute intoxication in the 1990s. Similar to the present cohort, the median age was 43 [29–60] years (p in comparison to present cohort = 0.442), and 52% were female (*p* = 0.095). The rate of pre-existing psychiatric disease was significantly lower with 49% of patients in this historic cohort, when compared to the 69% in our present study population (*p* < 0.0001). The rate of intubation and mechanical ventilation was significantly higher with 55% compared to 40% in the present (*p* < 0.0001). The median ICU LOS was 1 day (*p* = 0.596). The most common intoxication group was mixed intoxications, followed by the group of others and the group antidepressants, antipsychotics, or anticonvulsants. The most common reason for intoxication was suicidal intent in 52% of cases, followed by accidental in 14%, recreational in 10%, and iatrogenic causes in 1% of cases. In the historic cohort, the reason for the intoxication could not be identified in 23% of cases. In the 1990s, methods of primary poison or toxin removal were applied more often with 54% of cases predominantly using gastric lavage and activated charcoal (Table 2). Secondary removal techniques were used in 17% of cases mainly including hemodialysis, hemofiltration, and hemoperfusion. Nevertheless, overall ICU mortality remained stable over time with 4.2% in the 1990s and 4.1% in the present cohort.

## Discussion

In this study, we investigated all intoxicated patients who were admitted to our medical ICU over a 15-year-long period and compared the results to a historic 5-year cohort of the 1990s. We were able to show that mixed intoxications and suicidal intent were the most common intoxication group and reason.

Patients suffering from drug and substance abuse, as well as accidental and iatrogenic intoxications constitute a substantial part of the patient collective in medical ICUs worldwide. These patients, in the acute phase, oftentimes require airway, breathing and circulatory support, need constant observation and monitoring, as well as detoxification strategies including extracorporeal poison/toxin removal [10]. The types of intoxications and poisonings vary depending on the geographical region and are influenced by diverse local health policies and laws [11, 12]. In Europe, intoxication with barbiturates and pesticides is rare nowadays, but modern designer drugs may cause intoxications with previously unknown clinical presentation [13]. Adding to the heterogeneity of intoxications, those patients may be treated in outpatient facilities, emergency departments, general wards, intermediate care units, and ICU. The admission to ICU is based on the severity of disease, but may also depend on the availability of bed capacity or regional healthcare structures. Patients therefore may be admitted to the ICU solely for monitoring purposes, but also to receive full-code intensive care treatment including mechanical ventilation, antidotes, and extracorporeal poison or toxin removal.

The median age of patients in our study was 42 years with females being significantly older than males. In the present cohort, 75% of patients were younger than 55 years of age. These results are comparable to other studies with median ages of 45 years, with 83.1% of patients being younger than 70 years [3]. In a study by Kristinsson et al., the highest incidence of intoxication was in the groups of 20- to 49-year-olds [4]. Around half of the patients requiring ICU care stated that the reason for their poisoning was suicidal intent, and one-quarter of the patients indicated recreational usage as cause for their intoxication. The rate of suicidal intent was higher among females compared to men. There was a decline of intoxication with age and a shift from recreational intent in the youngest patients to suicidal intent in the mid-range regarding age to accidental intoxication in patients at higher age. To determine possible causes for the distribution of intoxication at a younger age and to develop mental health and addiction prevention measures, further research and advocacy is necessary. The results of our study are in line with several studies in the literature that found suicide, followed by recreational intent to be the most common reasons in ICU patients admitted for intoxication [14, 15]. Liakoni et al. found that 62.0% of patients presenting to the emergency department with illicit drug consumption were 21–40 years old [16]. Schwake et al. separated the group into adverse drug reactions which showed a median of 71 years of age, and intentional intoxications resulting in a median age of 39 years [17]. In our study, recreational intoxications were roughly four times more common in men than women, in line with a study also reporting that only every fourth recreational intoxication patient is female [18].

The prevalence of common substances utilized in acute intoxications varies broadly depending on the region’s socioeconomic status, availability of drugs, and local medication prescription practice and laws, as well as mental health and addiction measures that are put in place. As there is no accepted consensus on classification, we used a classification that has previously been published using nine categories to separate toxicological intensive care patients and to display the distribution of different intoxication cases [8]. Out of the nine categories, mixed drug intoxication was the most common intoxication group among both sexes. Thereafter, men were more likely to need ICU care following ethanol use, while females needed intensive care due to intoxication with antidepressants, antipsychotics, or anticonvulsants. Females had a higher rate of pre-existing psychiatric illness in our collective, which explains the easy access to substances in this group. In comparison to the historic cohort, the rate of psychiatric diseases increased; however, we cannot exclude coding changes or increased awareness to partially account for this increase.

Interestingly, the mortality of our collective was low despite being a high-risk selection of intoxicated patients, i.e., those that needed to be admitted to the ICU. We found an ICU mortality of 4.1% with no significant differences between sexes. Furthermore, mortality has not changed over time with 4.2% in our historic comparison group from the 1990s. The most lethal intoxication group was category 6 (carbon monoxide, arsenide, and cyanide) with 14.3%. Furthermore, those patients who died in the ICU had an exorbitantly poor prognosis already when arriving at ICU, as most of these patients had prolonged pre-hospital resuscitation. Our findings are therefore in the range of other investigations that found low mortality rates in the collective of intoxicated patients in the ICU. Liisanantti et al. reported a hospital mortality of 2.5%, while Siedler et al. and McMahon et al. had an ICU mortality of 4.4% and 6.3%, respectively [2, 3, 19]. However, some studies reported frankly high numbers of mortality, but this may be due to specific intoxications in some regions (e.g., organophosphorus) or scarcity of ICU beds, as only the sickest patients are admitted. Another explanation is that in some regions emergency medical personnel must transfer patients with ongoing CPR to the hospital, while in Austria emergency physicians are deployed pre-hospitally and resuscitations in patients with a futile prognosis are often terminated on scene. As a result, the data may differ from other emergency medical services, where different CPR termination protocols are in place. The University Hospital Graz is a central hospital offering a plethora of advanced and specialized fields and is the only public HBO center in Austria. Consequently, it is the only possible destination for patients suffering from severe carbon monoxide and smoke intoxications, therefore resulting in the admission and transfer of severely ill patients from other intensive care units. Risk factors associated with mortality in multivariable analysis in our study were intubation, elevated transaminases, and creatinine as well as low pH. These findings represent factors that are inherently associated with critical illness such as intubation, liver injury, and kidney failure. These factors were associated with worse outcomes in our study, but are neither sensitive nor specific for poisonings. Other factors that may contribute to higher mortality are elapsed time from exposure to intensive care admission and dose of exposure. Furthermore, the individual substances such as carbon monoxide contribute to mortality outcomes, as mentioned above. Liisanantti et al. found cardiovascular failure as a major risk factor for succumbing in the ICU [2]. Geith et al. found non-medical substances, low GCS, and others as predictors for fatal outcomes [20]. Nevertheless, one should not be fooled by the low mortality rate, as admission to ICU is often necessary and patients receive mechanical organ replacement therapy, airway protection, and poison elimination to survive the acute phase. However, due to the heterogeneity of methods, substances, delay to admission, and many other factors, it is difficult to define, apart from obvious reasons such as mechanical ventilation, clear criteria which patients may profit the most from ICU care. Thorough documentation of pre-hospital emergency medical services on last contact time, specific toxins or blisters found at the scene, and any other information on exposure time and dose may allow in future studies to better understand the influence of these factors on outcomes. Nevertheless, it may be possible that organ damage due to hypotension and hypoxemia are the main parameters determining mortality end points.

Primary removal techniques were performed in about 30% of cases and thus to a significantly lower extent than in the 1990s (54%). Initiation of a primary removal technique is considered useful within the first hour following ingestion of a substance, but patent airway reflexes or a protected airway with an endotracheal tube is mandatory [5, 21, 22]. Therefore, the possibility of activated charcoal administration is limited in patients with altered mental status and application requires a careful benefit to risk evaluation. One strategy to enhance the efficacy of primary poison or toxin removal may be to encourage adequate early pre-hospital administration of activated charcoal. Further research in intoxications with significant enterohepatic circulation or delayed gastric passage is necessary, as these groups may benefit more from this method of detoxification and even outside of the classic 1-h window of opportunity. Duineveld et al. found in an investigation from the Netherlands that 16.1–42.5% of intoxication patients received activated charcoal [23]. In another study from Germany published in 2021, only 10.9% of patients received a primary detoxification technique in the investigated years, but only ICU data were obtained [3], whereas we investigated all available data including emergency department, emergency medical services documentation, and ICU data. In our study, females received primary removal techniques more frequently than men. In this regard, it is possible that female patients had more ingested toxins or poisons, and might seek medical assistance earlier than men allowing for  activated charcoal treatment within the recommended time window. Furthermore, as noted before, men more often had ethanol and (intravenous) street drug intoxications, which cannot be treated with activated charcoal. Changes in clinical practice and international recommendations discouraged the use of induced emesis, gastric lavage, and whole bowel irrigation [24, 25]. This is also reflected by our data, as in the historic study cohort gastric lavage was used in 97% of all patients receiving a primary detoxification method, whereas in the present study population the application of these techniques significantly declined. In addition, despite the application of gastric lavage in the historic cohort the mortality rate remained stable over time, suggesting no benefit of this previous standard technique. In the repertoire of primary detoxification methods, the role of endoscopic medication retrieval is still uncertain, but has been applied especially in the context of pharmacobezoars [26].

Secondary detoxification methods were performed in 11.7% of cases. The term forced diuresis was used frequently in the medical documentation, but when applying our more stringent criteria forced diuresis was sparsely used. The multi-dose application of activated charcoal has been discussed in recent years and is only considered helpful in a minority of intoxications such as with carbamazepine, phenobarbital, quinine, theophylline, or dapsone [22, 27]. It was used more frequently in the recent than in the historic cohort, but we can only speculate on the reasons, as there were few indications on the individual patient level compared to the frequency of utilization of multi-dose activated charcoal. There was also a shift in the applied method of extracorporeal removal from hemoperfusion, hemodialysis, and hemofiltration in the historic cohort to hemodialysis and scarcely plasma exchange in the new cohort. Extracorporeal poison or toxin removal is an integral part of intensive care therapy and a life-saving procedure in selected intoxications. In addition, intoxications are a potential reversible cause in cardiac arrest and venoarterial extracorporeal membrane oxygenation may be used as a “bridge to recovery” [28]. Antidotes, and in particular flumazenil, were used frequently and maybe even uncritically by the treating physicians in the emergency medical services, the emergency department, or the ICU. However, flumazenil should not be used liberally, as severe adverse effects such as seizures and arrhythmias have been reported, especially in mixed intoxications with tricyclic antidepressants [29]. Other studies also have found high rates of antidote usage [15, 30], but further education on appropriate antidote usage is necessary. One specific treatment strategy in poisoned patients, the high insulin euglycemic therapy or HIET, was never used in our study cohort. However, it should be noted that it may be a lifesaving therapeutic option in severe calcium channel blocker or beta-blocker overdose.

### Strength and limitation

Other studies only used ICD codes to identify patients with intoxication in the ICU. This method has significant limitations, e.g., when another disease or a complication of the intoxication such as rhabdomyolysis or acute kidney injury was coded, but not the intoxication itself. In our study, we used both ICD codes and a full text search in the documentation of the patient to identify patients with potential intoxication and manually screened all documents for appropriateness. We were able to report the therapies from emergency physicians, emergency department and ICU, and obtained other necessary information from these documents not only relying on ICU documentation. We report real-life data of patients admitted to ICU; however, we did not prespecify criteria for ICU admission, as further studies are still needed to define patients that benefit significantly from ICU care. There is no clear consensus or definition for forced diuresis in literature. This global heterogenous terminology, however, makes it difficult to differentiate or compare the implementation of forced diuresis as a secondary detoxification method with other reports or publications. Interestingly, we noted that physicians used the term “forced diuresis” in their documentation heterogeneously. We did not assess for the appropriateness of the antidote application, and only investigated whether any antidote was given or not. This may result in a higher total number of antidotes given; however, the retrospective evaluation of only correct antidotes given would bias the results. The decision for application of antidotes must be performed at the bedside according to clinical assessment of the patients’ symptoms, often with little additional information available. This intrinsically leads to situations, where applied antidotes do not achieve  their intended effect. In addition, there is heterogenous use of the term antidotes in literature, as some substances, e.g., calcium gluconate or glucose, may be used for symptomatic treatment, but can also be used as a specific antidote in some poisoning. This limits the comparability between studies. Overall, a major limitation of our study is the single center design which may limit external validity.

## Conclusion

In our cohorts of poisoned patients admitted to ICU, the high rate of pre-existing psychiatric illness and the predominantly suicidal intent of intoxication imply that there should be a broad interdisciplinary, multimodal therapeutic approach to intoxicated patients with prompt referral to in- and outpatient psychiatric healthcare services. In addition, there are peculiar gender-specific aspects that must be considered in the critical care management of intoxicated patients, as females do present with a different spectrum of intoxication compared to males. Finally, the comparison with the historic cohort shows changes in the methods of primary detoxification, a growing subgroup of psychiatric diseases, and a generally low mortality of intoxicated patients over time.

## Supplementary Information

Below is the link to the electronic supplementary material.

Supplementary file1 (DOCX 53 KB)

## Data Availability

The datasets used and/or analyzed during the current study are available from the corresponding author on reasonable request.
