# Supplementary material for: Critical care management of acute intoxications, dynamics and changes over time: a cohort study
Source: Intern Emerg Med. 2024 Mar 19;19(7):2015–24. doi: 10.1007/s11739-024-03570-2 (PMC11466983; doi:10.1007/s11739-024-03570-2)
Supplement: Supplementary file 1 — Supplementary file1 (DOCX 53 KB) [file 11739_2024_3570_MOESM1_ESM.docx]

**Supplemental files**

**Critical care management of acute intoxications – dynamics and changes over time – a cohort study**

Alexander C. Reisinger, MD^1#^ (ORCID ID: 0000-0002-5773-5695); Nikolaus Schneider, MD^1#^; Nikolaus Schreiber, MD^1^; Martina Janisch, MD^1^; Ines Rauch, MD^1,2^; Peter Kaufmann, MD^1^; Gerrit Wünsch, Dr.rer.nat^3^; Philipp, Eller, MD^1*^; Gerald Hackl, MD^1^

#contributed equally

1. Department of Internal Medicine, Intensive Care Unit, Medical University of Graz, Graz, Austria
2. Department of Anesthesiology, LKH Hochsteiermark, Bruck an der Mur, Austria
3. Institute for Medical Informatics, Statistics and Documentation, Medical University of Graz, Graz, Austria

**Supplementary table 1: Applied keywords used in full-text search**

| **German Key Word** | **Translation in English** |
| --- | --- |
| Intoxikation | Intoxication |
| Vergiftung | Poisoning |
| Suizid | Suicide |
| Selbstmord | Suicide |
| akzidentiell | Accidental |
| suizidal | Suicidal |
| Toxikologie | Toxicology |
| Toxscreen | Toxic screening test |
| Screening | Screening |
| Drogen | Drugs |
| Rausch | High / drunk |
| Alkohol | Alcohol |
| Gift | Poison / venom |

**Supplementary table 2: ICD codes based on ICD10**

| **ICD code** | **Category name** |
| --- | --- |
| T36-T50 | Poisoning by drugs, medicaments and biological substances |
| T51-T65 | Toxic effects of substances chiefly nonmedical as to source |
| T96 | Sequelae of poisoning by drugs, medicaments and biological substances |
| F10.0 - F19.0 | Mental and behavioural disorders due to psychoactive substance use |
| 901, 902, 922, 923, 929, 931, 941, 999, U99.9 | Exogenous noxa - etiology |

ICD = International Classification of Diseases

**Supplementary table 3: Intoxication group and Circumstances leading to intoxication**

| **Intoxication pharmacological group** |  | **Circumstances leading to intoxication** |
| --- | --- | --- |
| unknown |  | unclear |
| ethanol |  | suicidal |
| analgesics |  | accidental |
| antidepressants, antipsychotics, anticonvulsants |  | recreational |
| street drugs |  | iatrogenic |
| sedatives |  |  |
| carbon monoxide, arsenic, cyanides |  |  |
| other toxins |  |  |
| mixed intoxications |  |  |

Intoxication pharmacological group based on Brandenburg et al (8) and circumstances of intoxication

**Supplementary table 4: List of psychiatric diagnoses present in patients of the study cohorts**

|  | Historical cohort | Present cohort |
| --- | --- | --- |
| Mental and behavioral disorders due to psychoactive substance use | 40 | 214 |
| Schizophrenia, schizotypal, delusional, and other non-mood psychotic disorders | 5 | 26 |
| Mood [affective] disorders | 44 | 190 |
| Anxiety, dissociative, stress-related, somatoform and other nonpsychotic mental disorders | 4 | 26 |
| Others | 1 | 36 |

Note that patients may be in more than one category. Classification was performed based on available information from medical records.

**Supplementary table 5: Antidotes used in the study cohort**

| flumazenil |
| --- |
| naloxone |
| acetylcysteine |
| physostigmine |
| glucose |
| glucagon |
| biperiden |
| digitalis antidote |
| toluidine blue |
| methylene blue |
| silymarin |
| phylloquinone |
| pyridoxin |
| snake antivenom |
| ethanol in toxic alcohols |
| atropine in betablocker intoxication |
| dantrolene |
| prothrombin complex concentrate |
| calcium in calcium channel blocker poisoning |
| folic acid |
| sodium hydrogencarbonate in tricyclic antidepressant poisoning |
| sodium thiosulfate |
| fomepizole |
| hydroxocobalamin |

Note that we did not assess for the appropriateness of the antidote application, and only investigated whether any antidote was given or not. However, we only counted antidotes that were used to treat the specific poisoning and not used for symptomatic treatment in other indications such as glucose for hypoglycemia outside the context of insulin overdose.
